# Defining parasite biodiversity at high latitudes of North America: new host and geographic records for *Onchocerca cervipedis* (Nematoda: Onchocercidae) in moose and caribou

**DOI:** 10.1186/1756-3305-5-242

**Published:** 2012-10-30

**Authors:** Guilherme G Verocai, Manigandan Lejeune, Kimberlee B Beckmen, Cyntia K Kashivakura, Alasdair M Veitch, Richard A Popko, Carmen Fuentealba, Eric P Hoberg, Susan J Kutz

**Affiliations:** 1 Department of Ecosystem and Public Health, Faculty of Veterinary Medicine, University of Calgary, 3330 Hospital Drive NW, Calgary, AB, T2N 4N1, Canada; 2 Canadian Cooperative Wildlife Health Centre, Calgary AB Canada, Faculty of Veterinary Medicine, University of Calgary, 3330 Hospital Drive NW, Calgary, AB, T2N 4N1, Canada; 3 Division of Wildlife Conservation, Alaska Department of Fish and Game, 1300 College Road, Fairbanks, AK, 99701, USA; 4 Department of Environment and Natural Resources, Government of the Northwest Territories, Norman Wells, NT, Canada; 5 Present address: Department of Structure and Function, School of Veterinary Medicine, Ross University, Basseterre, St. Kitts, St. Kitts and Nevis; 6 US National Parasite Collection, Agricultural Research Service, USDA, BARC East No. 1180, 10300 Baltimore Avenue, Beltsville, MD, 20705, USA

**Keywords:** *Alces*, Caribou, Legworm, North America, *Onchocerca cervipedis*, Moose, *Rangifer*, Subarctic, Vector-borne diseases

## Abstract

**Background:**

*Onchocerca cervipedis* is a filarioid nematode of cervids reported from Central America to boreal regions of North America. It is found primarily in subcutaneous tissues of the legs, and is more commonly known as ‘legworm’. Blackflies are intermediate hosts and transmit larvae to ungulates when they blood-feed. In this article we report the first records of *O. cervipedis* from high latitudes of North America and its occurrence in previously unrecognized host subspecies including the Yukon-Alaska moose (*Alces americanus gigas)* and the Grant’s caribou (*Rangifer tarandus granti*).

**Methods:**

We examined the subcutaneous connective tissues of the metacarpi and/or metatarsi of 34 moose and one caribou for parasitic lesions. Samples were collected from animals killed by subsistence hunters or animals found dead in the Northwest Territories (NT), Canada and Alaska (AK), USA from 2005 to 2012. Genomic DNA lysate was prepared from nematode fragments collected from two moose. The *nd5* region of the mitochondrial DNA was amplified by PCR and sequenced.

**Results:**

Subcutaneous nodules were found in 12 moose from the NT and AK, and one caribou from AK. Nematodes dissected from the lesions were identified as *Onchocerca cervipedis* based on morphology of female and male specimens. Histopathological findings in moose included cavitating lesions with multifocal granulomatous cellulitis containing intralesional microfilariae and adults, often necrotic and partially mineralized. Lesions in the caribou included periosteitis with chronic cellulitis, eosinophilic and lymphoplasmacytic infiltrate, and abundant granulation associated with intralesional adult nematodes and larvae. Sequences of the *nd5* region (471bp), the first generated for this species, were deposited with Genbank (JN580791 and JN580792). Representative voucher specimens were deposited in the archives of the United States National Parasite Collection.

**Conclusions:**

The geographic range of *O. cervipedis* is broader than previously thought, and extends into subarctic regions of western North America*,* at least to latitude 66°N. The host range is now recognized to include two additional subspecies: the Yukon-Alaska moose and Grant’s caribou. Accelerated climate change at high latitudes may affect vector dynamics, and consequently the abundance and distribution of *O. cervipedis* in moose and caribou. Disease outbreaks and mortality events associated with climatic perturbations have been reported for other filarioids, such as *Setaria tundra* in Fennoscandia, and may become an emerging issue for *O. cervipedis* in subarctic North America.

## Background

*Onchocerca cervipedis* Wehr & Dikmans, 1935 (Nematoda; Onchocercidae), is a widespread filarioid parasite of cervids, reported from Costa Rica to boreal regions of North America [1-4]; it is also considered as a typical parasite in cervids across the Holarctic [5]. It was first described in white-tailed deer (*Odocoileus virginianus)* and black-tailed deer (*Odocoileus hemionus columbianus*) from Montana and British Columbia in 1935 [6]. A filarioid found in the pronghorn (*Antilocapra americana*) in 1934 was subsequently assigned to this species [7], and there have since been several reports in species of *Odocoileus*[8-11] throughout Canada and the USA (excluding Alaska), and a single report in white-tailed deer from Costa Rica, Central America [1]. It has also been reported in the wapiti (*Cervus canadensis*) from Idaho [8]. Published reports of *O. cervipedis* in moose have been restricted to the subspecies *Alces americanus andersoni* in British Columbia [12], southern Alaska [4] and northern Alberta [2,13]. Gross lesions consistent with infection, however, have been observed in Yukon-Alaska moose (*Alces americanus gigas*) from the Yukon (Philip Merchant, pers. comm.). A single report in *Rangifer* is from a woodland caribou (*Rangifer tarandus caribou*) from Tweedsmuir Park, BC [3].

*Onchocerca cervipedis* generally affects subcutaneous tissues of hindquarters from the tibio-tarsal joint to hoof, and thus is more commonly known as ‘legworm’ or ‘footworm’. The presence of *O. cervipedis* rarely cause clinical signs; however, massive infections can cause swelling and hoof damage in species of *Odocoileus*, which may increase susceptibility to predation [8,9]. Clinical disease has not been reported in caribou, nor has associated histopathology been described from any of its multiple hosts.

Blackflies (Diptera: Simuliidae) act as intermediate hosts (IH), transmitting the parasite to ungulates during blood meals. Studies on black fly vectors of this filarioid are scarce in the literature. In California, *Prosimulium impostor* is the only known IH responsible for deer infection [14]. *Simulium decorum* and *Simulium venustum* are demonstrated as IH in northeastern Alberta, and other simuliid species have been reported feeding on moose; however, their competence as IH are not known [13].

Disease emergence associated with climatic conditions and new host associations has been reported for related arthropod-borne filarioids of ungulates at higher latitudes. For example, peritonitis epizootics caused by *Setaria tundra* in reindeer were linked to unusually warm climatic conditions in Fennoscandia [15,16]. Emergence of *Onchocerca skrjabini* (*= O. tarsicola*) in reindeer (*Rangifer tarandus*) in Sweden during the late 60’s was hypothesized to be caused by range expansion of the putative primary host, the red deer (*Cervus elaphus*) see [17]. Under current conditions of climate warming, shifts in distribution of suitable vector species and more favourable temperatures for both parasite and vector development and survival, have led to the geographic expansion and/or amplification in endemic areas of various arthropod-transmitted pathogens. Both phenomena can result in disease emergence [16,18-21]. Therefore, a better understanding of the diversity, host and geographic range, and ecology of these parasites is warranted.

Passive and active disease surveillance efforts, together with hunter-based wildlife health monitoring efforts [22] from 2005 to 2012, led to the discovery of *O. cervipedis* in moose and caribou across higher latitudes of Canada and Alaska. The objective of this paper is to report on these discoveries and present associated molecular and histopathological findings.

## Methods

### Sampling

**Figure 1:**
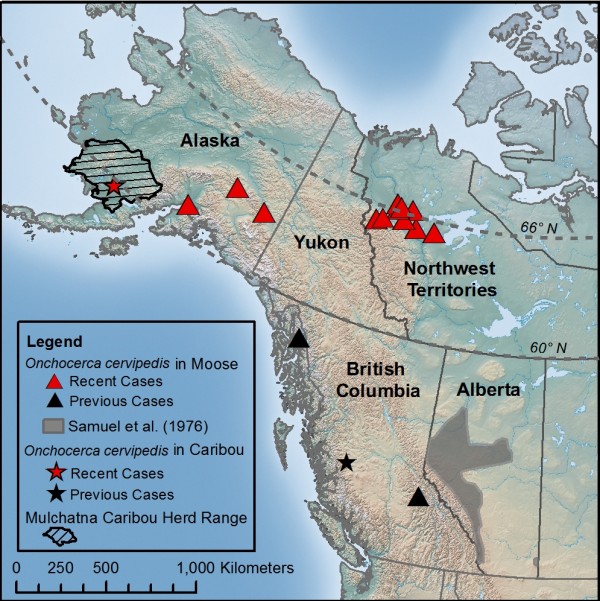
**Reports of *O. cervipedis *from the present study and previous northernmost reports in northwestern North America.**

*Opportunistic collections*: Between 2005 and 2012, incidental nodular lesions on the metacarpus and/or metatarsus were detected and collected during skinning of hunted moose (*A. a. gigas* and *A. a. andersoni*) from the Northwest Territories (NT), Canada, and Alaska (AK), and caribou (*R. t. granti*) sampled during the Mulchatna herd health survey by the Alaska Department of Fish and Game (Table 1). *Alces a. gigas* is the moose sub-species which ranges in AK, Yukon and the Mackenzie Mountains, NT and has a short seasonal migration of up to 196km [see [23]. *Alces a. andersoni* is distributed throughout central Canada, and north-central USA, extending north through much of the boreal, taiga and tundra areas of mainland NT, and parts of Nunavut. Individuals of this subspecies do not tend to migrate and have overlapping home ranges [24]. In contrast, caribou of the Mulchatna herd range over 69,457 km^2^ (range = 54,337–78,513 km^2^, 1997–1998 estimate) in southwestern AK (Figure 1) and have an annual seasonal migration between winter and summer range [25]. 

**Table 1:** **Records of *O. cervipedis *in moose and caribou from the Northwest Territories and Alaska**

**Host/Animal ID**	**Host subspecies**	**Date collected**	**Age class/Sex**	**Herd/Location**	**Coordinates**	**Accession numbers**	
						**USNPC**	**GenBank**
**Moose - AK**							
2005-176	*Alces a. gigas*	Sept, 2005	Adult male	Tok River, East Central AK	63°10'00.47"N 143°05'09.75"W^§^	104120	-
2006-185	*Alces a. gigas*	Sept, 2006	Adult male	Palmer area, Southcentral AK	61°24'39.96"N 150°19'22.80"W	104119	-
2011-131	*Alces a. gigas*	Sept, 2011	Adult	Salcha, Interior AK	63°10'00.47"N 143°05'09.75"W^§^	105387	-
**Moose - NT**							
UC187	*Alces a. andersoni*	June, 2007	Adult male	Kelly Lake, NT	65°26'7"N 126°10'40"W^§^	104778	JN580791
W76-09Ta	*Alces a. gigas*	Sept, 2009	Adult male	Tabasco Lake, NT	65°17'00''N 131°7'7''W	103495	JN580792
W76-09Tb	*Alces a. gigas*	Sept, 2009	Calf	Tabasco Lake, NT	65°17'00''N 131°7'7''W	103495	-
WT-05	*Alces a. andersoni*	Dec, 2010	Adult	Ramparts River, NT	66°06'20"N 129°01'17"W^§^	N/A	-
WT-21	*Alces a. andersoni*	Nov, 2010	Adult	Apache Pass, NT	65°43'16"N 127°53'32"W^§^	105702	-
WT-06	*Alces a. andersoni*	Jan, 2011	Adult	Hare Indian River, NT	66°16'12"N 127°12'00"W^§^	105701	-
WT-81	*Alces a. andersoni*	Feb, 2011	Adult	Willow Lake, NT	65°28'10"N 123°57'35"W^§^	105703	-
WT-60	*Alces a. andersoni*	Jan, 2012	Adult	Near Fort Good Hope, NT	66°15'0"N 128°37'0"W^§^	105779	-
WT-65	*Alces a. andersoni*	Jan, 2012	Adult	Tidia River, NT	66°22'30"N 129°11'W^§^	105780	-
**Caribou**							
2006-061	*Rangifer t. granti*	June, 2006	Adult female	Mulchatna herd, near Kaliganek, Southwestern AK	59°42'27.50"N 157°18'36.33"W^§^	100427	-

^§^ Approximate geographic coordinates.

Submitted metacarpi and metatarsi were examined for lesions and these were dissected and nematodes collected, frozen, and subsequently preserved in 70% ethanol for morphological and molecular identification. A subset of tissues containing lesions was preserved in 10% formalin and processed routinely for histopathological examination.

*Moose health survey (Sahtu, NT):* Between 2010 and 2012, during a subsistence hunter-based health survey on moose from the Sahtu Settlement Area, NT, the left leg (metatarsus to hoof) on each animal was collected to evaluate body condition (marrow bone). Subcutaneous tissues of the metatarsals from a total of 28 moose, 26 from the Fort Good Hope area and 2 from Deliné area were examined for lesions (Table 1)*.* Tissues containing lesions were collected and examined using a dissecting microscope.

### Parasitological examinations

Female and/or male nematodes collected from moose were cleared in lactophenol or phenol-alcohol, examined microscopically, and identified according to the literature for the species [6,26] and compared with all other *Onchocerca* species infecting cervids [27-30]. Tissues from one of the Tabasco Lake adult moose (W76-09Ta) and the Mulchatna caribou were prepared for histology: embedded in paraffin, sectioned and subsequently stained with haematoxylin-eosin.

Genomic DNA (gDNA) lysate was prepared from nematode fragments collected from Kelly Lake moose and Tabasco Lake moose. Briefly, fragments of 2-4mm were individually transferred into 0.2mL tubes containing 5μL of deionized water. To each tube was added 50μL of lysis buffer (0.4mg/mL of proteinase K) and these were incubated at 60°C for 60 min, followed by 95°C for 15 min. DNA lysate was diluted 1:20 in DNase RNase free ddH_2_O. PCR was performed using primers ND5OvA (5'-TTGGTTGCCTAAGGCTATGG-3') and ND5OvC (5'-CCCCTAGTAAACAACAAACCACA-3') targeting the *nd5* region of mitDNA, using a protocol modified from the literature [31]. PCR amplification was performed in 25μL reactions containing 16.7μL of water, 2.5μL of 10x PCR buffer, 2.0 μL of MgCl_2_, 0.5μL of 10mmol dNTPs, 0.5μL (40pmol) of each primer, 0.3μL of *Taq* DNA polymerase, and 2μL of DNA template. Amplification conditions consisted of an initial 2min denaturation at 95°C, followed by 35 cycles of 95°C for 1min, 50°C for 45s, and 72°C for 30s. A final extension of 72°C for 5min was followed by cooling to 4°C. Reagent-only reactions were used as negative controls.

PCR products were sequenced directly using ND5OvA and ND5OvC primers using BigDye Terminator Cycle Sequencing (Applied Biosystems). Sequences were edited using FinchTV 1.4.0 and MEGA version 5 [32]. BLAST searches were used to compare the resulting sequences to those available in GenBank.

## Results

*Onchocerca cervipedis* were recovered from subcutaneous nodular lesions from 12 moose in Alaska and NT and one caribou in Alaska (Table 1, Figure 1). Lesions compatible with those caused by *O. cervipedis* were found in 21.4% (6/28) of the examined moose legs from the Sahtu Settlement Area, NT, subsistence hunt and nematodes were found in five nodules out of six examined.

**Figure 2:**
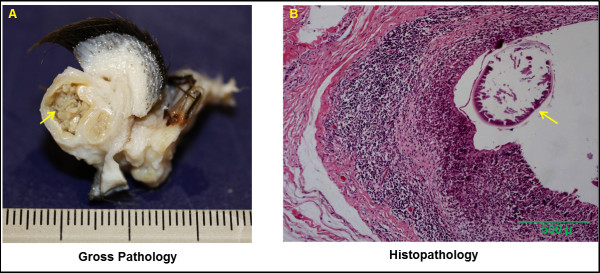
**Gross and histologic changes in a case of *O. cervipedis *infection in a moose (W76-09Ta) from Northwest Territories, Canada. A**) Cut surface of a subcutaneous nodule surrounded by a thick connective tissue capsule and containing a yellowish, friable material and intralesional *O. cervipedis* adult nematodes (arrow); **B**) Histological transverse section of a partially mineralized dead adult nematode within a cavitating lesion lined by multinucleated giant cells, macrophages, eosinophils, lymphocytes and plasma cells. HE stain (100x).

**Figure 3:**
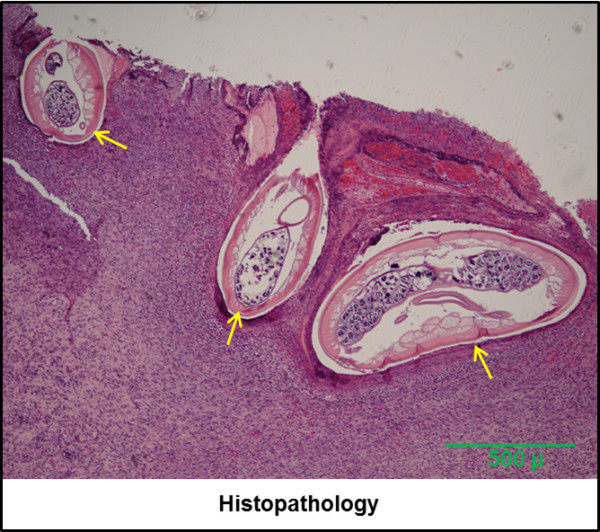
**Histopathologic features of a case of *O. cervipedis *infection in a caribou from Alaska, USA.** Histological section of periosteum and subcutaneous tissue nodular lesion demonstrating adult female nematodes (arrows) characterized by presence of a cuticle with thick transverse ridges and coelomyarian musculature, embedded in granulation tissue. HE stain (100x).

Characteristic gross lesions could only be observed after the metatarsals were skinned. Grossly, subcutaneous nodules were firm, generally round to ovoid but a few were indistinct in form, and ranged from 2-5mm in length. On the cut surface, a yellowish friable material and filarioid nematodes were observed, surrounded by thick fibrous connective tissue. Histologically, in moose there were cavitating lesions with multifocal granulomatous cellulitis containing intralesional microfilariae and adults. These were characterized by presence of cuticle with thick transverse ridges and coelomyarian musculature and were often necrotic and partially mineralized (Figure 2a,b). Material from caribou demonstrated chronic periosteitis and cellulitis, eosinophilic and lymphoplasmacytic infiltrate (Figure 3). The inflammatory infiltrate was composed of numerous eosinophils, lymphocytes, and plasma cells admixed with prominent neovasculation and fibroplasia, interpreted as granulation tissue.

Nematodes were identified as *O. cervipedis,* based on morphological characteristics of female (cuticular pattern, vulva-head distance, esophagus length) and male (spicule length and ratio) nematodes. Specimens were deposited in the US National Parasite Collection, USDA (Table 1; Figure 1).

Sequences of the *nd5* region of the mitDNA (471bp) were the first generated for *O. cervipedis* and were deposited in GenBank under accession numbers JN580791 and JN580792 (Table 1). These two sequences differed between each other by a single nucleotide (99% similarity). BLAST comparisons revealed 88-93% of similarity with sequences from other species within the genus *Onchocerca*.

## Discussion

### Distribution

Our findings demonstrate a considerably broader geographic range for *O. cervipedis,* extending into subarctic western North America*,* at least to latitudes of 66°N. Together with anecdotal reports of *Onchocerca* sp. in moose from the Yukon Territory (P. Merchant, Pers. Comm.), our results suggest a continuous distribution of *O. cervipedis* in the northern boreal forest regions of western North America. Additionally, we provide two new host records for *O. cervipedis*: the Yukon-Alaska moose (*A. a. gigas*) and Grant’s caribou (*R. t. granti*), supporting contentions that this parasite is a host generalist [1-11]. Migratory behaviour of some of the host species (e.g., Mulchatna caribou and Alaskan moose), may facilitate range expansion across sometimes vast distances. Due to the opportunistic nature of our sampling and the small sample size we could not evaluate age or sex predilections. In general, adult ungulates are reported to be more frequently infected [13]; however, calves and fawns are rarely harvested and this may have biased previous conclusions.

The impact of *O. cervipedis* in moose and caribou remain unknown. Clinical disease attributed to *O. cervipedis* in deer is most commonly confined to distal limbs and is associated with pain, loss of digits and hoof, swelling, and ulceration with female nematodes protruding through the lesions [8,9,33]. Such pathology would certainly affect animal mobility, and interference in normal activities may make clinically affected animals easier prey to both predators and hunters [8]. In contrast, we found *O. cervipedis* to be fairly common in moose and its occurrence may be characterized as an incidental finding with minimal pathology in ‘healthy’ moose harvested for subsistence at higher boreal latitudes of North America. The nodules in moose and the caribou were typically found along the metatarsus or metacarpus, and caused a mild to moderate local inflammatory reaction. In these hosts, parasite localization may also affect locomotion but the distal limbs and joints are not affected. There are, however, a few reports of clinical disease, with *Onchocerca* sp. associated with open sores on the legs of moose in the Yukon (Philip Merchant, Pers. Comm.). In general, detection of sick animals in the wild may be difficult. Heavily parasitized animals with reduced mobility may be rapidly eliminated by wolf and bear predation, and therefore, the significance and extent of *O. cervipedis* in northern Canada and Alaska is difficult to establish.

### Related *Onchocerca* species

Until recently, *O. cervipedis* was thought to be the only *Onchocerca* species parasitizing native cervids of North America. Molecular characterization of *Onchocerca* microfilariae from subcutaneous tissues of white-tailed deer in Northeastern USA revealed that at least one other species is present in native North American cervids [34]. In contrast, five *Onchocerca* species have been described infecting wild cervids in the Palearctic [27]. This is consistent with a general pattern of greater nematode species diversity in ungulates of Eurasia. This pattern is related to the historical expansion and geographic colonization of ungulate hosts and parasites, including filarioids, from Eurasia into North America over the past 2–3 Myr [e.g., [35]. However, recent studies in North America further suggest that the biodiversity of *Onchocerca* species from the Nearctic has been underestimated, and deserves more rigorous investigation combining comparative morphological and molecular approaches.

Regarding the broader diversity for species of *Onchocerca* across the Holarctic, *O. cervipedis* is reported from Sakha and the Altai region of Russia [see [5], although conspecificity of Eurasian and North American parasites remains to be determined. Among Palearctic species in cervids, *Onchocerca alcis* has been described from tendon insertions of the tibia in European moose, and seems to be most closely related to *Onchocerca jakutensis (= O. tubingensis),* a red deer (*Cervus elaphus*) parasite [27]*.* Other species that infect red deer are *O. skrjabini, Onchocerca flexuosa*, and *Onchocerca garmsi*. *Onchocerca skrjabini* is commonly found in reindeer in Sweden. It does not form nodules but is found free in tissues surrounding the tendons of tibio-tarsal and radio-carpal joints, and also in other parts of the body, including muzzle and shoulders [17]. Similarly, *O. garmsi* does not form nodules and it can be found free in subcutaneous tissues of the sternal region. *Onchocerca flexuosa* is found in subcutaneous nodules on the back, chest, and abdomen of red deer [36]. The nodular lesions caused by the Nearctic *O. cervipedis* in moose and reindeer seem to be more similar to those caused by *O. flexuosa. Onchocerca flexuosa*, however, has also been associated with necrotic foci in the liver, and to a lesser extent in kidney, myocardium, and other tissues in slaughtered Swedish reindeer. Histologically, these foci or granulomas contain larval or adult nematodes, and culture revealed *Corynebacterium* spp., thus constituting a relevant meat hygiene issue and resulting in discard of affected organs see [17]. A more systemic distribution of *O. cervipedis* has not been observed nor has the potential for secondary bacterial infection been investigated. If found this may be of food safety relevance for subsistence and other hunters.

### Parasite and vector ecology

The only Simuliidae known to be involved in the transmission of *O. cervipedis* to moose in northern Alberta were *Simulium decorum* and *Simulium venustum*[13]*.* Both *S. decorum/noelleri* (as they are morphologically indistinguishable) and species within the *S. venustum* complex are present in the Mackenzie Mountains, NT [37,38]. Other species that feed on moose and other large ungulates, such as those in the *Simulium arcticum* complex and *Simulium vittatum/tribulatum,* also occur in the study area in the NT (D. Currie, unpublished data), but their competency as vectors for *O. cervipedis* is unknown. The above mentioned species and/or species-complex are also widely distributed in Alaska, and the Yukon [39,40]. In California, *Prosimulium impostor* is thought to be the only dipteran vector responsible for transmission of *O. cervipedis* to Columbian black-tailed deer [14]. Although *P. impostor* is not reported in our study area, a variety of other species within the genus *Prosimulium* are present [37,38].

Filarioid nematodes may be particularly sensitive to climatic changes both through effects on vector abundance and parasite development. In Finland, severe disease outbreaks in reindeer caused by *S. tundra* have been linked to episodes of unusually warm climatic conditions [15,16,19]. Under current climate warming scenarios for northern Canada and Alaska [41] we might anticipate substantial changes in the ecology, distribution, and abundance and impacts of *O. cervipedis,* and perhaps other filarioids, in northern ungulates [e.g., [20,42]. Climate-facilitated range expansion of the parasite may become of particular importance for naive migratory tundra caribou populations. To date, *O. cervipedis* has not been reported in these caribou, and was not found by our group despite examination of metatarsals from over 500 barrenground caribou (*R. t. groenlandicus*) across North America between 2007 and 2011 [43]. Sitka black-tailed deer (*O. hemionus sitkensis*), potentially sympatric with some moose populations in Alaska, may also serve as suitable definitive hosts and should be further investigated.

Some species of *Onchocerca* parasites of wild cervids are zoonotic. For instance, *Onchocerca jakutensis,* infecting red deer (*Cervus elaphus*) in Europe, has been the cause of nodular dermatological disease in humans [44]. This raises the possibility of zoonotic potential in other *Onchocerca* species associated to wild ungulates, such as *O. cervipedis.*

## Conclusions

Climate warming is known to influence distribution and transmission dynamics of many parasitic organisms, in particular those requiring arthropod or other ectothermic species as vectors or intermediate hosts to complete their life-cycles. Our study, based on opportunistic sampling, together with the recent discovery of a potentially new species of *Onchocerca* in white-tailed deer in the USA, demonstrates that knowledge of species distribution and diversity in North America is incomplete. Similarly, the ecology and impacts of *O. cervipedis* in general, and particularly at northern latitudes, are poorly understood. These gaps in knowledge pose important limitations on our ability to anticipate how climate change may affect these host-parasite interactions in subarctic regions and the potential for northern range expansion into *R. t. groenlandicus* populations at arctic latitudes.

## Competing interests

The authors declare no competing interests.

## Authors' contribution

GGV lead the study and preparation of the manuscript. SJK oversaw the study and manuscript preparation. GGV and EPH did morphological identification of specimens. GGV and ML carried out the molecular genetic study, CKK, AMV, RAP, and SJK participated in acquiring data from the moose health survey in the NT. KBB, SJK, AMV, RAP, and CF acquired specimens and provided histopathologic findings from moose and caribou material from Alaska and Canada. All authors critically revised and approved the final manuscript.
